# Deep learning for video-based automated pain recognition in rabbits

**DOI:** 10.1038/s41598-023-41774-2

**Published:** 2023-09-06

**Authors:** Marcelo Feighelstein, Yamit Ehrlich, Li Naftaly, Miriam Alpin, Shenhav Nadir, Ilan Shimshoni, Renata H. Pinho, Stelio P. L. Luna, Anna Zamansky

**Affiliations:** 1 Information Systems Department, University of Haifa, Haifa, Israel; 2 School of Veterinary Medicine and Animal Science, São Paulo State University (UNESP), São Paulo, Brazil; 3 Faculty of Electrical Engineering, Technion, Israel Institute of Technology, Haifa, Israel; 4 Faculty of Veterinary Medicine, University of Calgary, Calgary, Canada

**Keywords:** Animal behaviour, Machine learning

## Abstract

Despite the wide range of uses of rabbits (*Oryctolagus cuniculus*) as experimental models for pain, as well as their increasing popularity as pets, pain assessment in rabbits is understudied. This study is the first to address automated detection of acute postoperative pain in rabbits. Using a dataset of video footage of n = 28 rabbits before (no pain) and after surgery (pain), we present an AI model for pain recognition using both the facial area and the body posture and reaching accuracy of above 87%. We apply a combination of 1 sec interval sampling with the Grayscale Short-Term stacking (GrayST) to incorporate temporal information for video classification at frame level and a frame selection technique to better exploit the availability of video data.

## Introduction

Rabbits (*Oryctolagus cuniculus*) are widely used worldwide as experimental models, especially in translational research on pain. They also are increasingly popular as pets, who may experience various painful conditions^1^. However, research on pain assessment in rabbits is still underdeveloped^2^. For instance, despite the high number of rabbits undergoing surgical procedures, the protocols for anesthesia and analgesia in rabbits are still limited compared to those for cats and dogs^3^.

One method for pain assessment is behavioral assessment which is simple, multidimensional, noninvasive, painless, does not require physical restraint and allows remote assessment, providing easy pain assessment in investigation and medical settings. To our knowledge there are four behavior-based scales to assess acute postoperative pain in rabbits. The Rabbit Grimace Scale (RbtGS)^4^ is a facial expression based scale developed and evaluated in rabbits submitted to ear tattooing. Another scale developed to assess pain in pet rabbits by merging this facial scale with physiological and behavioural parameters is the composite pain scale for rabbit (CANCRS)^5^. Another two scales with more robust validations are the Rabbit Pain Behavioral Scale (RPBS) to assess postoperative pain^6^ and the Bristol Rabbit Pain Scale (BRPS)^7^.

A recent systematic review by Evangelista et al.^8^ assessed evidence on the measurement properties of grimace scales for pain assessment, addressing internal consistency, reliability, measurement error, criterion and construct validity, and responsiveness of the grimace scales. The Rabbit Grimace Scale (RbtGS) only exhibited moderate level of evidence (as opposed, e.g., to mouse or rat grimace scales that were found to be of high level of evidence). Moreover, the scoring is affected by various factors, such as procedures the animal is subjected to, environment^9^, and perhaps most importantly, are susceptible to bias and subjectivity of the human scorer. An additional limitation of the RbtGS is the presence of cage bars that rabbits are frequently housed in, which can compromise the assesment of facial expressions. This leads to the need for the development of more precise methods for scoring and assessing pain in rabbits which are less susceptible to these factors.

Automated recognition of pain is addressed by a large body of research, several reviews focus on facial expression assessment in humans^10^, and, specifically in infants^11^. For animals, on the other hand, this field has only emerged a few years ago, but is now growing rapidly. Broome et al.^12^ review over twenty studies addressing non-invasive automated recognition of affective states in animals, mainly focusing in pain. It is highlighted that the problem of noisy facial analysis for animals is even more challenging than in humans due to technical and ethical challenges with data collection protocols with animal participants, but perhaps more importantly, the challenges with data quality due to obstruction, blurred images due to movement, challenging angles, cage bars, among others. Species that have been addressed in the context of automated pain recognition include rodents^13–15^, sheep^16^, horses^17–19^, cats^20,21^ and dogs^22^.

To the best of our knowledge, this is the first study addressing automated detection of acute postoperative pain in rabbits. Using a dataset of video footage of n = 28 rabbits before (no pain) and after surgery (pain), we developed an AI model for pain recognition using both facial area and body posture, reaching accuracy of above 87%. The second, more technical contribution of this study is addressing the problem of information loss in static analysis, i.e., working with frames (as opposed to videos). As highlighted in Broome et al.^23^, static analysis is the simplest and least expensive option in terms of computational resources, and indeed almost all the works on pain recognition reviewed in Broome et al.^23^ opt for this path. However, this implies information loss: as was demonstrated in Broome et al.^18^ for horses, dynamics is important for pain recognition. The alternative of working with video data directly, however, as reported in^18,24^, requires computationally heavy training, and is extremely data-hungry, requiring data in volumes that we did not have in our dataset.

To address the problem of information loss, we propose a two-step approach that utilizes sequences of frames. Our method applies a combination of 1 sec interval sampling with the Grayscale Short-Term stacking (GrayST) to incorporate temporal information for video classification at frame level. After training a ’naive’ model with sampled and stacked frames, we apply a frame selection technique that uses confidence levels of our ’naive’ pain classifier. This approach significantly improves performance, reaching above 87% accuracy, while using a much smaller dataset of better quality. Our proposed method provides a practical solution for pain recognition, enabling accurate analysis without sacrificing computational efficiency.

## Results

For narrative purposes we preface our results with essential and practical aspects to improve understanding for those less familiar with AI methods, presenting a high-level overview of the used approaches, as well as with the dataset description.

### Overview

**Figure 1:**
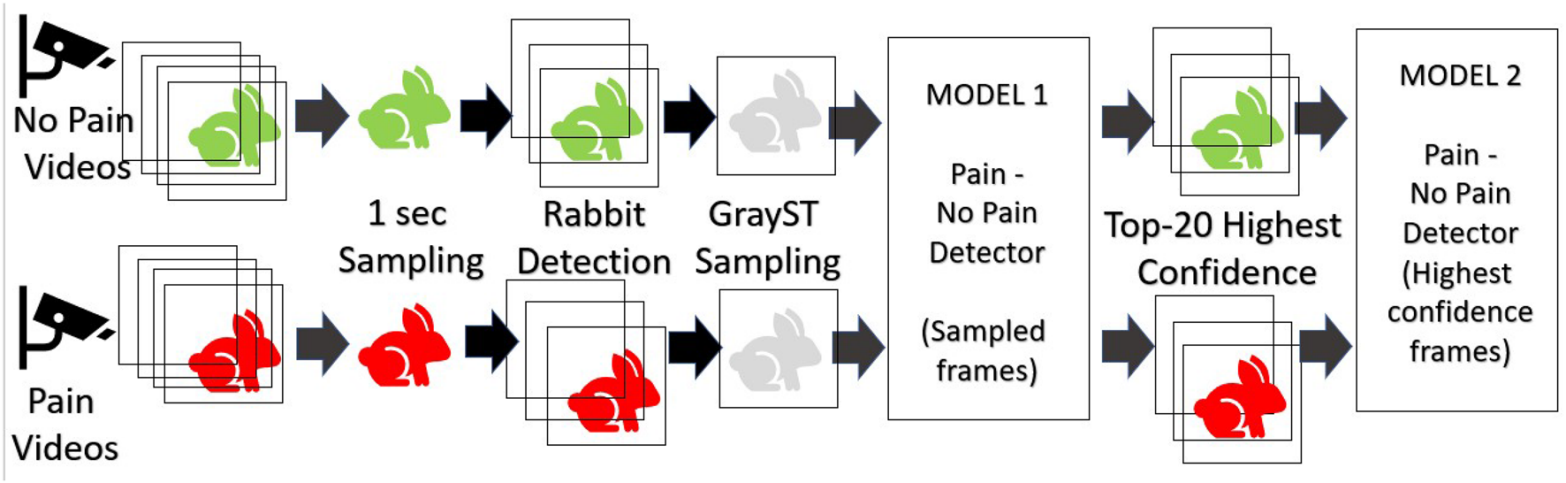
Pipeline description.

Figure 1 presents a high-level overview of the two-staged pipeline used in this study. At the pre-processing stage, rabbits are automatically detected (using Yolov5 object detection model) and cropped, and videos are sampled, extracting a single frame every second. As a second step, samples are converted to grayscale and aggregated using GrayST stacking method. Then the first model is trained on all sampled frames. We then use confidence levels (how “sure” the model is of its classification of a frame) to choose the top n = 20 frames for each class (pain/no pain). The intuition here is by this specific manner of undersampling we can remove ‘noisy’ frames caused by the in-the-wild videos containing many low-quality frames, due to obstruction (bars, rabbit not facing camera), blurry frames (caused by movement), or the fact that pain level reflected visually does not always remain on the same fixed level throughout the video. Such removal of ‘noise’ indeed leads to increased performance of the second model which is trained only on the top (highest confidence) frames.

**Figure 2:**
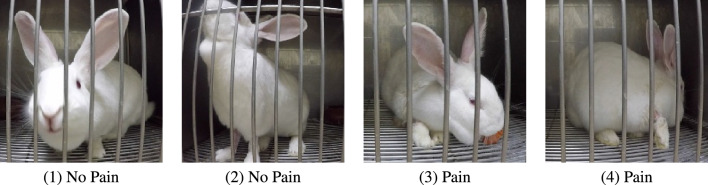
Example of cropped frames.

### Dataset

We used a portion of the video dataset of Haddad et al.^6^, collected for the aim of validation of the rabbit pain behaviour scale (RPBS) to assess acute postoperative pain in rabbits, which captured rabbits undergoing orthopaedic surgery (the Ortho dataset of Haddad et al.^6^). This dataset was collected during a study that was approved by the Ethical Committee for the Use of Animals in Research, of the School of Veterinary Medicine and Animal Science and School of Agricultural and Veterinary Sciences, São Paulo State University (Unesp), under protocol numbers 0156/2018 and 019155/17, respectively. The study follows the Brazilian Federal legislation of CONCEA (National Council for the Control of Animal Experimentation); University of Haifa waived further ethical approval. The dataset includes footage corresponding to pre/post-operative periods of 28 rabbits (11 females and 17 males) that were recorded at different time points corresponding to varying intensities of pains during surgery process: ‘baseline’ (before surgery), ‘pain’ (after surgery, before analgesic), ‘analgesia’ (after analgesic), and ‘24h post’ (24 h after surgery). Overall, the footage contained 112 videos of 2–3 minutes length. Four rabbits showing RPBS scale score equal or above pain threshold (3) during ’baseline’ stage were excluded. For our final dataset we selected 48 videos with one video labeled as ‘No Pain’ (before surgery stage) and one video labeled as ‘Pain’ (after surgery) for each of the 24 individuals, leading to a balanced dataset of overall 24 videos for each class (pain/no pain). Figure 2 shows examples of frames from both ‘pain’ and ‘no pain’ classes.

### Model Performance

For measuring the performance of the models, we use standard evaluation metrics of accuracy, precision, recall and F1 (see, e.g., Lencioni et al^17^ for further details).

As a validation method^25^, we use leave-one-subject-out cross validation with no subject overlap. Due to the relatively low numbers of rabbit (n = 24) and samples (n = 24 * 2) in the dataset, following the stricter method is more appropriate^15,18^. In our case this means that we repeatedly train on 19 subjects, validate on 4 and test on the remaining subject; Table 1 presents the aggregated average result. By separating the subjects used for training, validation and testing respectively, we enforce generalization to unseen subjects and ensure that no specific features of an individual are used for classification.

Table 1 displays the performance outcomes of a pipeline we experiment with two different backbones: ResNet50 and CLIP/ViT + Naive Bayes. In both cases we performed the following two phases.

1. Naive phase. The initial model, referred to as “ Model 1” was trained first on all frames and did not utilize GrayST pre-processing. This model, employing the Resnet50 transfer learning architecture, achieved an accuracy of 66% and 69% employing CLIP + Naive Bayes backbone. However, when GrayST pre-processing was employed, the model’s performance improved to 77% and 81% employing CLIP + Naive Bayes backbone.
2. Improved phase. The next “Model 2”, on the other hand, was only trained on the frames with the highest confidence (obtained in “Model 1”), achieving an improved accuracy of 83% with Resnet50 backbone and 87% employing CLIP + Naive Bayes backbone. On both type of backbones, “Model 2” trained on the frames with the highest confidence exhibited the best performance. Note that to test “Model 2” (in both Resnet and CLIP cases) we use the same strict cross-validation method of leave-one-subject-out to avoid ofer-fitting. “Model 2” is tested on all frames of videos belonging to rabbits taken out for testing.

Aggregating from single frames to video prediction, we average confidence levels (pain and no-pain scores) for each class and selecting the class with the highest average, similar to the Average Pooling method described in^26^. The aggregated results are presented in Table 1, which displays the video classification results using combinations of training sets consisting of all frames or only Top frames, and using or not using the GrayST aggregation method.

An interesting by-product of the frame selection process described above should be noted. Table 2 shows the performance of both types of classifiers (ResNet and CLIP) using only datasets obtained from the selected top frames (for both classes). The fact that “Model 2” used on selected top frames performs so much better than on (all frames on all ) videos reflects the presence of more informative signals of pain in these top frames, essentially yielding an automated method for frame selection which could replace manual selection of frames from videos employed, e.g., in^20,21^.

**Table 1:** Video classification performance comparison.

		Resnet50 transfer learning				CLIP VIT B/32 Encoding + Gaussian Naive Bayes			
Train set	GrayST	Accuracy	Recall	Precision	F1	Accuracy	Recall	Precision	F1
Model 1 (all frames)	No	0.66	0.54	0.72	0.61	0.69	0.59	0.74	0.66
Model 1 (all frames)	Yes	0.77	0.66	0.84	0.74	0.81	0.67	0.94	0.78
Model 2 (top frames)	Yes	0.83	0.83	0.83	0.83	0.87	0.87	0.87	0.87

**Table 2:** Top frames image classification performance comparison.

Model	Accuracy	Recall	Precision	F1
Resnet50 transfer learning	0.93	0.96	0.93	0.94
CLIP VIT B/32 Encoding + Gaussian Naive Bayes	0.96	0.96	0.96	0.96

**Figure 3:**
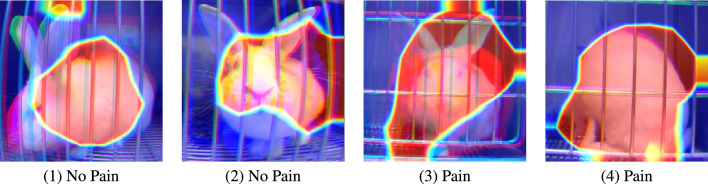
Examples of GradCAM applied to TOP frames.

## Discussion

To the best of our knowledge, this work is the first to address automation of post-operative pain recognition in rabbits. The ‘naive’ model trained on *all* frames reached accuracy of above 77% using the technique of GrayST. It should be noted that our dataset contains noisy video footage of subjects appearing in different angles in cages with bars. The used method of frame selection manages to reduce ‘noise’ in this data, with performance increasing to above 87%.

As expected, the accuracy of 87% reached here for rabbit pain recognition outperforms the approaches of^18,22,24^, which work with video and are comparable to previous work for automated pain detection with frames^17,20,21^. The benefit of the frame selection approach used here is not only in increasing accuracy, but also dealing with occlusion and a variety of angles of rabbit in a cage. In our experiments we compared the performance of the Resnet50-based architecture to the more novel CLIP VIT-based architecture. As can be seen in Table 1, the latter exhibits a slightly superior performance. These rather similar performance results emphasize the contribution of the proposed pipeline disregarding the very different model architectures used for the pain classification.

The outstanding performance of Vision Transformer (ViT) models in identifying pain in rabbits, as well as other emotional states such as positive anticipation and frustration in dogs^27^, is indicative of their superiority. This can be attributed to several factors, such as their enhanced attention mechanism that enables ViT models to capture long-range dependencies and focus on relevant image features related to pain. Furthermore, ViT models consider global contextual information, which aids in recognizing subtle cues across the entire image. Their deeper architecture and larger number of parameters also provide a higher representational capacity, enabling them to capture fine-grained details associated with pain. Transfer learning from large-scale pretraining on image datasets further enhances their performance by providing a strong initial understanding of visual concepts, which can be effectively generalized to the pain identification task. These factors collectively contribute to the improved performance of ViT models in identifying pain in animals.

The models used in this study are deep learning models, which means that they are ‘black-box’ in their nature. As discussed in^21^, one common approach to explore explanability of such models is to apply visualization methods that highlight the areas in the image that are of importance for classification. We applied the GradCAM (Gradient-weighted Class Activation Mapping)^28^ technique to the Top images obtained from our model. Figure 3 shows some examples. These examples demonstrate that the model focuses on facial areas in certain images, while in others, attention is directed towards body areas. This observation prompts further investigation into the regions exploited by the models to discern pain, as well as the importance of body posture as opposed to facial expressions in machine pain recognition. A more systematic investigation of explainability of the obtained models along the lines of^21^ is an immediate future direction.

Moreover, when training and testing using only with selected Top images Table 2 shows a superior accuracy of even 95% which indicates that such subset of selected images contains high valuable information about pain and may be useful for researchers to investigate what it seen in those images, combined with previously described visualization techniques. The results obtained in rabbit pain recognition are highly promising, with an accuracy of 87%, which outperforms previous approaches that used video, such as^18,22,24^. This method is also comparable to previous work for automated pain detection with frames^17,20,21^. The approach used here, which involves selecting frames, not only increases accuracy but also deals effectively with occlusion and a variety of angles of the rabbit in a cage.

**Figure 4:**
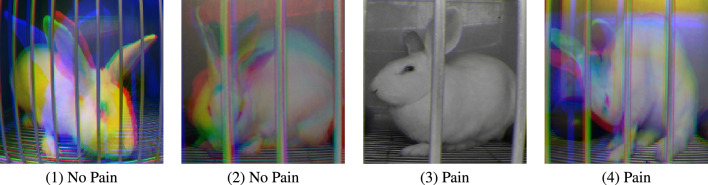
Examples of cropped frames after Grayscale Short-Term Stacking (GrayST).

The utilization of a combination of techniques, namely 1-sec sampling and the Gray-ST aggregation of three frames into a single frame, has been found to significantly enhance the capacity of pain detection models. This improvement can be attributed to the fact that reduced animal movement is considered a behavioral indicator of pain, as described in RPBS, and the temporal information related to this indicator appears to be more effectively captured by the combined implementation of these techniques. For example, Fig. 4(3) displays a complete gray image of a rabbit without any colored area. Such image indicates that during three consecutive seconds this rabbit remained static, which correlates with a painful state. However, further analysis is necessary to comprehend the effectiveness of distinct sampling intervals on the performance of pain identification. Given that temporal information seems to be a major factor for pain identification, it is recommended that temporal models be further investigated.

It is important to note that while some elements of the developed approach are rabbit-specific most of the elements can be reused across species. In particular, we have tested the GrayST and the frame selection techniques studied here on the dataset from^20^, and achieved increased performance. It seems that the pipelines can be reused for various species after some fine-tuning (e.g., the cropping pre-processing is species-specific).

## Methods

### Preprocessing

*1. Trimming and frame sampling*. Videos contain large amounts of temporally redundant data, making it possible to skip some parts without losing much information^29^. Assuming that pain expressions may be intermittent but with a certain continuous duration over time, we trimmed every 2-min length video, selecting one frame per second. Every video was recorded using a 60 frames per second encoding. Thus each video was reduced from 7200 frames (60 frames/s $\times$ 120 s) to 120 frames.

*2. Rabbit Detection and Cropping*. We customized a Yolov5^30^ object detector using a manually annotated dataset with 179 rabbit images, extracted from the original dataset. A total of 142 images of different individuals were used for training the detector, and 37 for validation. Using the rabbit detector, images were cropped, focusing on the rabbit.

**Figure 5:**
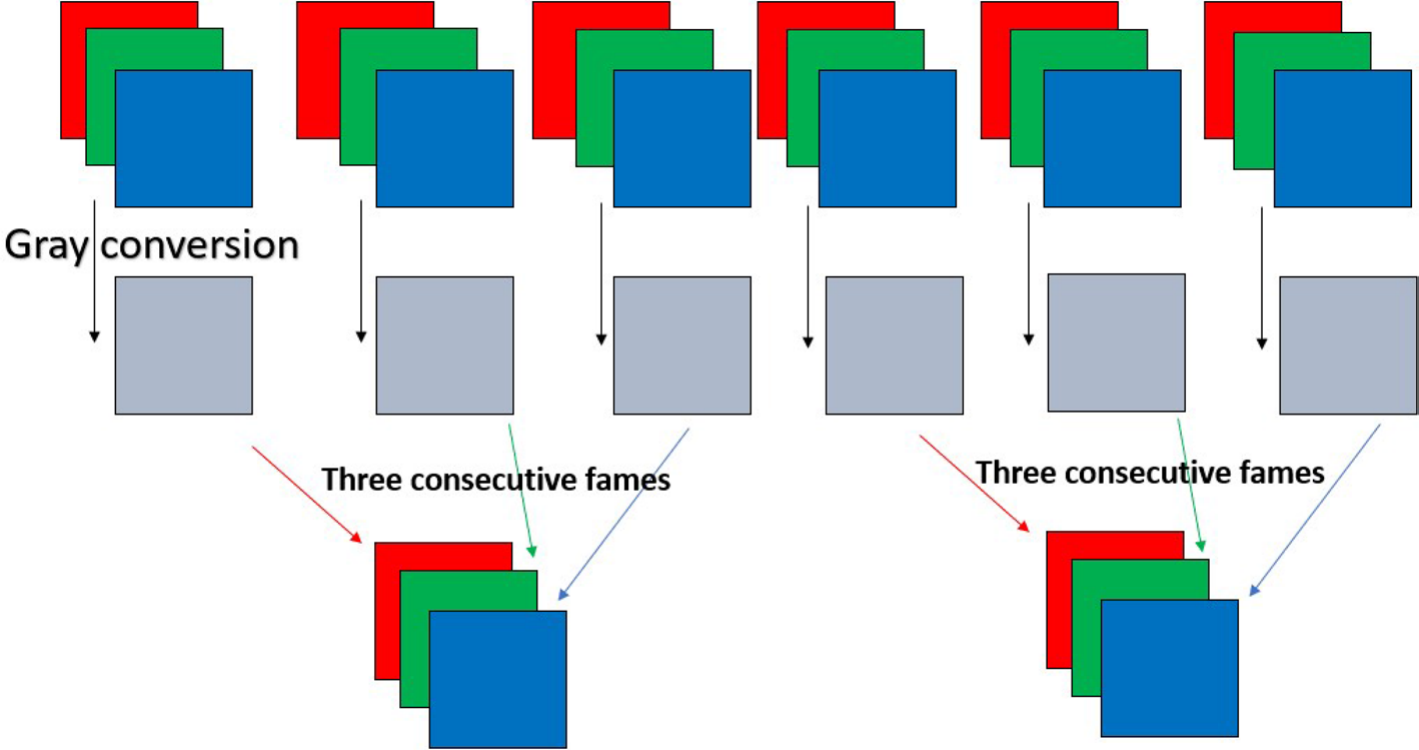
Grayscale Short-Term Stacking (GrayST) preprocess.

*3. Grayscale Short-Term Stacking (GrayST)*. We use the Grayscale Short-Term Stacking (GrayST), a methodology proposed in^31^, to incorporate temporal information for video classification without augmenting the computational burden. This sampling strategy involves substituting the conventional three color channels with three grayscale frames, obtained from three consecutive time steps. Consequently, the backbone network can capture short-term temporal dependencies while sacrificing the capability to analyze color. A description of GrasyST process is shown in Fig. 5. Figure 4 shows examples of frames from both ‘pain’ and ‘no pain’ classes after application of Grayscale Short-Term Stacking (GrayST).

### Model training

We investigated two different types of deep learning pipelines: Transfer Learning using a pretrained Resnet50 architecture and CLIP embedding combined with Gausian Naive Bayes Classification.

#### Transfer Learning using a pretrained Resnet50

Similarly to^32^, we apply transfer learning on a Resnet50 model pre-trained on ImageNet, provided in the Tensorflow package for Keras using ImageNet weights without its head. On top of the last layer, we added a new sub network compound of an average pooling layer, a flatten layer, a fully connected (FC) layer of 128 cells, a 0.5 dropout layout and a softmax activation layer for pain/no pain categorization. The model was compiled using binary cross-entropy loss and Adam optimizer, and all layers in the base model were set as non-trainable to retain the pre-trained weights during the initial training phase. We used batch size of 64 with a learning rate of 1e-4, and chose the model that achieved the best (maximal) validation accuracy. Every image was augmented applying only changes on the image size or illumination like a random zoom range of up to 0.15, width shift of up to 0.2, height shift of up to 0.2, shear range of up to 0.15. We did not apply any augmentation that may change the angle of the image since we assumed important visual information could be contained in body position changes.

#### CLIP embedding combined with Gausian Naive Bayes classification

CLIP^33^ encoding is a process of mapping images into a high-dimensional embedding space, where each image is represented by a unique embedding vector. The CLIP encoder achieves this by pre-training a neural network on a large dataset of image and text pairs using a contrastive loss function. In this work, we encode images using a ViT-B/32 architecture, a specific instance of a Vision Transformer (ViT) model that can be used as an image encoder in CLIP. The “ViT” in ViT-B/32 stands for Vision Transformer, “B/32” refers to the batch size used during training of the model. It indicates that during the training process, the data is divided into batches, with each batch containing 32 samples. Batch size is an important parameter in machine learning models and affects the efficiency and memory requirements during training. We extract the output of the final layer as a 512 dimensional embedding vector that will be used for pain classification.

The Naive Bayes classification model^34^ is a probabilistic algorithm used for classification tasks in machine learning. It is based on Bayes’ theorem, which describes the probability of a hypothesis given some observed evidence. The “naive” assumption in the model is that the features used to represent the data are independent of each other, which simplifies the probability calculations. The model estimates the probability of each class given the input features and then assigns the input to the class with the highest probability. Naive Bayes is computationally efficient and can work well even with small amounts of training data.

### Frame selection

We used the obtained classification models (ResNet Model 1 and CLIP/ViT Model 1) to select N Top frames with the highest confidence to train their corresponding Model 2 (ResNet Model 2 and CLIP/ViT Model 2 respectively). In the ResNet-based model, after the last layer added on top of the pre-trained model, we used the binary entropy values of two classes (no pain, pain) as confidence values of the Resnet50 model. For the Gaussian Naïve Bayes classifier we used with the CLIP model, the confidence level is the probability estimation for the test vectors (image embeddings).

For our experiments, we chose N = 20. The intuition here is by this specific manner of undersampling we can remove ‘noisy’ frames caused by the in-the-wild videos containing many low-quality frames, due to obstruction (bars, rabbit not facing camera), blurry frames (caused by movement), or the fact that pain level reflected visually does not always remain on the same fixed level throughout the video. Such removal of ‘noise’ may lead to increased performance of the model, thus we experimented by using only the top-20 frames data for training another model.

**Figure 6:**
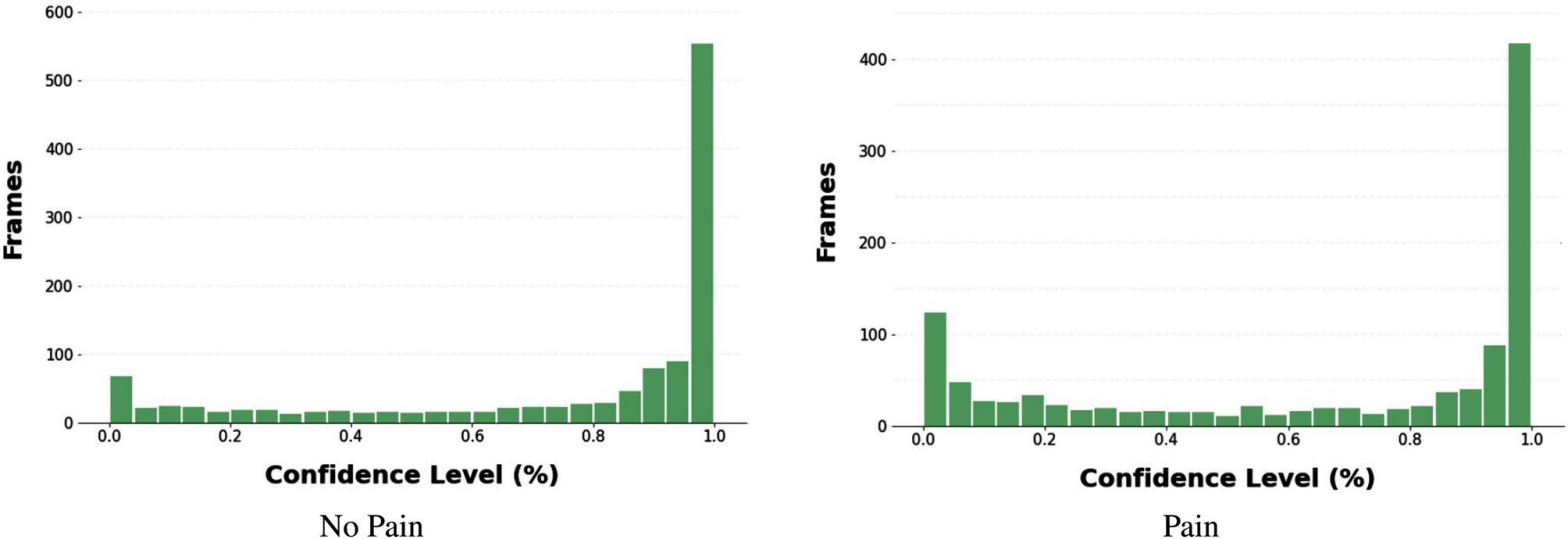
Confidence histogram of frames classified by Resnet50 model.

**Figure 7:**
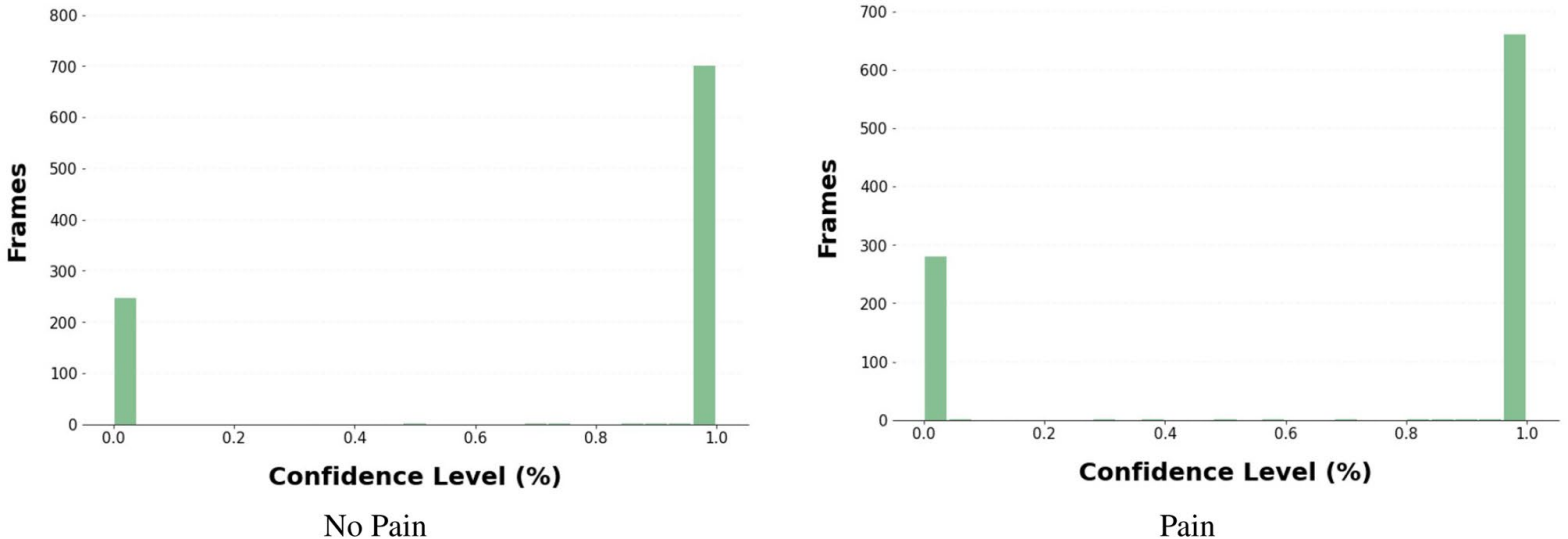
Confidence histogram of frames classified by CLIP model.

Figures 6 and 7 show the confidence level distributions of frames classified by the Resnet50 and CLIP/ViT models respectively, with the majority of frames with high confidence levels. Our new Top-20 dataset consists of 20 images of pain and 20 images of no pain for each rabbit. The exact same training procedure as described above was used for training new models using the Top-20 dataset.

## Data Availability

The dataset is available from the corresponding authors upon request.
